# Astrovirus VA1 identified by next-generation sequencing in a nasopharyngeal specimen of a febrile Tanzanian child with acute respiratory disease of unknown etiology

**DOI:** 10.1038/emi.2016.67

**Published:** 2016-07-06

**Authors:** Samuel Cordey, Francisco Brito, Diem-Lan Vu, Lara Turin, Mary Kilowoko, Esther Kyungu, Blaise Genton, Evgeny M Zdobnov, Valérie D'Acremont, Laurent Kaiser

**Affiliations:** 1 Laboratory of Virology, Infectious Diseases Service, University of Geneva Hospitals, Geneva 1211, Switzerland; 2 University of Geneva Medical School, Geneva 1211, Switzerland; 3 Department of Genetic Medicine and Development, University of Geneva Medical School, Geneva 1211, Switzerland; 4 Swiss Institute of Bioinformatics, Geneva 1211, Switzerland; 5 Amana Regional Referral Hospital, Dar es Salaam PO BOX 25411, United Republic of Tanzania; 6 Tanzanian Training Centre for International Health, Ifakara PO BOX 39, United Republic of Tanzania; 7 Swiss Tropical and Public Health Institute, University of Basel, Basel 4002, Switzerland; 8 Department of Ambulatory Care and Community Medicine, University of Lausanne, Lausanne 1011, Switzerland; 9 Infectious Disease Service, University Hospital, Lausanne 1011, Switzerland

**Dear Editor,**

Over the past decade, next-generation sequencing (NGS) has become a useful tool to identify novel or emerging infectious agents.^1^ Although NGS is not yet used as a routine diagnostic tool, it is increasingly used as a second line of investigation for select clinical cases, particularly when routine molecular and serological assays fail to identify a causative pathogen. We developed a NGS-based pipeline (ezVIR) to process NGS data for the identification of viruses in humans^2^ with the aim of identifying divergent or unexpected viruses in biological specimens of subjects with unexplained clinical syndromes.^2, 3^

We used ezVIR to analyze 30 nasopharyngeal swab specimens collected in a previous study that examined the causes of fever in Tanzanian children^4^ who were suffering from respiratory symptoms of any type but for whom an infectious agent was not detected (except colonizing bacteria in the nasopharynx) despite extensive investigations, including viral, bacterial and parasitic molecular and serological assays.^4^ The specimens were pooled in groups of five and assessed according to the specific RNA and DNA library preparation protocols as previously described^2^ for NGS analysis (paired-end sequencing using the 100-bp protocol with indexing on a HiSeq 2500 (Illumina, San Diego, CA, USA). Unexpectedly, an astrovirus VA1 was identified in pool 1 (Figure 1; Supplementary Table S1).

Each specimen in pool 1 (patients T32, T794, T359, T560 and T524) was analyzed individually using the astrovirus VA1-specific real-time reverse-transcription polymerase chain reaction (RT-PCR) targeting the capsid region (forward primer 5′-CCA TCA GCA GTT ACY GGG TCT GT-3′ reverse primer 5′-CGT GGC TCC AGG TGA YTG T-3′ probe 5′-FAM-TTT CCG CAT ATC CC-MGB_NFQ-3′) under the following cycling conditions: 50 °C for 30 min; 95 °C for 15 min; 45 cycles of 15 s at 94 °C and 1 min at 55 °C. Patient T359 was confirmed positive for astrovirus VA1 (Ct value=29.6; Figure 1). The initial T359 nasopharyngeal specimen was reanalyzed individually by NGS. NGS data were analyzed with ezVIR and completed by performing a de novo analysis. As shown in the ezVIR phase 2 report, 91.5% coverage of the astrovirus VA1 genome was obtained (305 mapped reads), with a maximum coverage depth of 11-fold. Phylogenetic analysis of the capsid fragment consensus sequence (1077 nucleotides long) obtained from *de novo* assembly^5^ shows the closest nucleotide homology (97.8% Figure 1) with the novel human astrovirus (HAstV) VA1/HMO-C-UK1 sequence (KM358468, corresponding to nucleotides 5137−6213). The presence of a parainfluenza virus type 4 (PIV-4) was also detected in the ezVIR phase 2 report, with lower signals for both parameters (174 mapped reads, 38.5% genome coverage, maximum coverage depth of sevenfold). However, using the FTD Respiratory pathogens 21 commercial assay (Fast-track Diagnostics, Sliema, Malta), the PIV-4 genome was not detected in the specimen, suggesting a viral load below the limit of detection of this assay (the limit of positivity was Ct values ⩽37). By contrast, in agreement with the ezVIR phase 2 report for pool 1 (Figure 1), patients T32 and T794 were confirmed positive for PIV-2 (Ct value=21.6) and PIV-4 (Ct value =31), respectively, by specific real-time RT-PCR. This finding suggests that the PIV-4-negative result in patient T359 reflects a very low viral load rather than potential mismatches between the assay target and the viral genome sequences of some PIV-4 circulating in this specific area during the study. For this reason, PIV-4 is considered unlikely to be responsible for the respiratory symptoms in this patient. Furthermore, an intrinsic consequence of highly sensitive NGS technology is the increased detection of interspecimen contamination. Therefore, the presence of several thousands of PIV-4 mapped reads in pool 1 and pools 4, 5 and 6 allows the possibility of potential contamination from neighboring PIV-4-positive samples.

Patient T359 was a 13-month-old girl brought to the Amana District Hospital in Dar es Salaam, Tanzania, in June 2008 for fever (39.3 °C), runny nose and cough for the past three days. She had no other symptoms, in particular no diarrhea, abdominal pain, vomiting or rash. Except for the elevated temperature, the patient's vital signs were normal (the respiratory rate was 35 breaths/min), there was no sign of severe sepsis or respiratory distress, and the detailed physical examination did not reveal any abnormality. The caregivers responded negatively to questions about the presence of acute disease in contact persons or chronic disease in the child. The leukocyte count (leukocytes 33 × 10^9^/L with a neutrophilic predominance), C-reactive protein (139.1 mg/mL) and procalcitonin levels (5.772 μg/L) were elevated. Other blood tests were within the normal range (hemoglobin 9.7 g/dL, alanine transaminase 43 U/L, hematocrit 29.1% and platelets 453 × 10^9^/L). The urine dipstick was positive for leukocytes, and thus the patient was treated with cotrimoxazole, but the urine culture was sterile. As mentioned, no viral, bacterial or parasitic agent was evidenced after a broad range of investigations, including blood culture. In particular, virological screening included the following specific real-time RT-PCR assays: influenza A and B; respiratory syncytial virus; metapneumovirus; PIV types 1 and 3; rhinovirus; enterovirus; coronavirus (HKU1, NL63, 229E and OC43); bocavirus; adenovirus; human herpesvirus 6; parvovirus B19; dengue; chikungunya; West Nile and Rift Valley viruses. She was also screened for antigen detection of adenovirus and rotavirus, and serological assays targeting IgM for Epstein−Barr virus, cytomegalovirus, hepatitis A and E, mumps and measles were performed. The clinical diagnosis retained was a tracheobronchitis of unknown etiology.

Astrovirus VA1 belongs to the novel *Mamastrovirus* 9 species (*Astroviridae* family) and was first identified in 2009 in human stools during an outbreak of acute gastroenteritis.^6, 7, 8^ Its prevalence in stools ranges from 0.2% to 1.6% in diarrheic and non-diarrheic subjects worldwide.^8, 9, 10^ Furthermore, astrovirus VA1 has been identified in brain biopsies of immunocompromised patients with central nervous system disease,^9, 11, 12, 13^ but it has never been documented in respiratory specimens until now. Current molecular diagnostic assays used in most routine laboratories target only the classical HAstV 1–8 serotypes, which belong to the *Mamastrovirus* 1 species; yet, astrovirus VA1 cannot be diagnosed because it shares only a 46% nucleotide sequence identity with the full genome of HAstV-1. This is particularly unfortunate, as a recent investigation of healthy adult blood donors in the United States showed that 65% of the samples were seropositive.^14^ In terms of public health relevance, there is increasing evidence that astrovirus VA1 and other novel HAstVs recently described worldwide (i.e., astrovirus VA1-5 and MLB1-3^15^) may be of importance in routine diagnostics, particularly in immunocompromised patients. To our knowledge, our finding is the first documented case of an astrovirus VA1 in the respiratory tract, and it justifies considering the potential association of recently identified new human astroviruses, such as astrovirus VA1, with respiratory diseases of unknown origin. Large prospective prevalence investigations are needed for a better understanding of the epidemiological and clinical relevance of novel astroviruses.

## Figures and Tables

**Figure 1:**
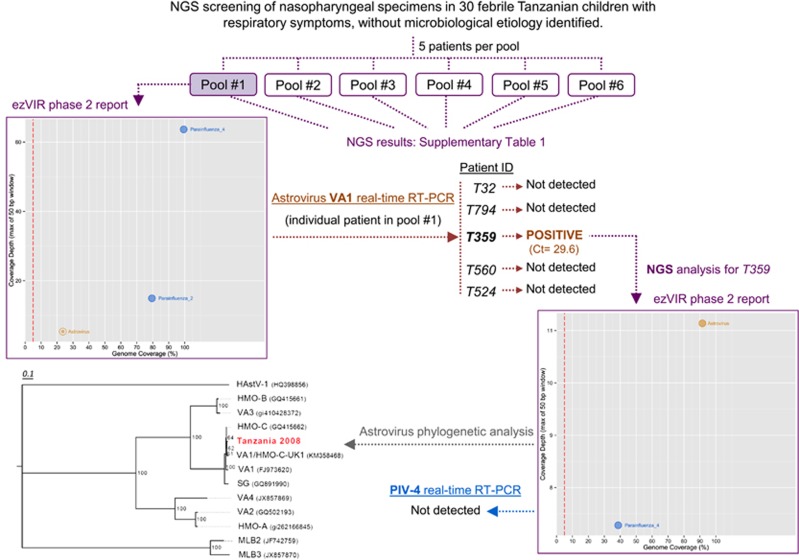
Specimen analysis flowchart. NGS analyses are in purple. Astrovirus VA1 and PIV-4-specific real-time RT-PCR screenings are in orange and blue, respectively. The phylogenetic analysis is based on a 1077-nucleotide-long fragment that maps to the capsid region (nucleotides 5137 to 6213 of VA1/HMO-C-UK1 (KM358468)). Abbreviations: cycle threshold, Ct; next-generation sequencing, NGS; parainfluenza virus type-4, PIV-4.
